# A novel adaptive trial design: randomised evaluation of molecular guided therapy for diffuse large b-cell lymphoma with bortezomib (REMODL-B) with two interim analyses to explore safety and efficacy

**DOI:** 10.1186/1745-6215-14-S1-O77

**Published:** 2013-11-29

**Authors:** Tom Maishman, Louise Stanton, Andy Davies, Sharon Barrans, Lisa Worrillow, Christoph Mamot, Matt Care, Tikki Immins, Debbie Hamid, Andrew McMillan, Paul Fields, Andrew Jack, Peter Johnson

**Affiliations:** 1 University of Southampton Clinical Trials Unit, Southampton, Hampshire, UK; 2 Cancer Research UK Centre, Southampton General Hospital, Southampton, Hampshire, UK; 3 Haematological Malignancy Diagnostic Service (HMDS), St. James's Institute of Oncology, Leeds, UK; 4 Centre for Oncology, Haematology and Transfusion Medicine, Kantonsspital Aarau, Tellstrasse, Switzerland; 5 Section of Experimental Haematology, Leeds Institute of Molecular Medicine, Leeds, UK; 6 Department of Haematology, Nottingham City Hospital, Nottingham, UK; 7 Department of Haematology, Guy's Hospital, London, UK

## Background

Retrospective molecular profiling of untreated Diffuse Large B-Cell Lymphoma (DLBCL) samples has recognised distinct sub-classifications of this disease, each with unique biological features and clinical outcomes.

## Objectives

(i) To demonstrate superior clinical efficacy, of bortezomib in combination with rituximab and CHOP (RB-CHOP) versus R-CHOP for the treatment of previously untreated patients with DLBCL.

(ii) To assess whether the molecular phenotype determines benefit from the addition of bortezomib.

## Methods

The trial uses an adaptive design and aims to recruit 940 patients across 100 sites in the UK and Switzerland (currently recruited 398 patients). After being profiled within real time (during cycle 1), ABC, GCB and unclassifiable molecular phenotype patients are randomised to receive RB-CHOP or R-CHOP with equal allocation. Two interim analyses will be carried out to stop the good prognosis GCB group early if required using a Case Morgan [1] analysis:

(i) The first will be a safety analysis performed after the first 55 GCB RB-CHOP patients have been followed for 6 months. If 6 month PFS<80%, the GCB group will close.

(ii) The second will be for futility in the GCB group and performed after the first 73 GCB RB-CHOP patients have been followed for 1 year. If 1 year PFS<85%, the GCB group will close.

If the GCB group closes the trial will then be modified to a randomised Phase II trial in ABC patients and sample size calculations will be revised.
